# Magnesium supplementation enhances insulin sensitivity and decreases insulin resistance in diabetic rats

**DOI:** 10.22038/ijbms.2020.40859.9650

**Published:** 2020-08

**Authors:** Hongzhou Liu, Nan Li, Mengmeng Jin, Xinyu Miao, Xinjie Zhang, Wenwen Zhong

**Affiliations:** 1 Department of Endocrinology, First Hospital of Handan City, No. 25 Congtai Road, Handan, Hebei Province 056002, China; 2 Department of Endocrinology, The Second Medical Center & National Clinical Research Center for Geriatric Diseases, Chinese PLA General Hospital, No. 28 Fuxing Road, Beijing 100853, China; 3 Healthcare Department, The Second Medical Center & National Clinical Research Center for Geriatric Diseases, Chinese PLA General Hospital, No. 28 Fuxing Road, Beijing 100853, China

**Keywords:** Insulin receptor, Insulin sensitivity, Magnesium, Metabolic diseases, Type 2 diabetes

## Abstract

**Objective(s):**

Diabetes mellitus has been suggested to be the most common metabolic disorder associated with magnesium deficiency. This study aimed to investigate the effects and mechanisms of magnesium supplementation on insulin receptor activity in elderly type 2 diabetes using a rat model and to provide experimental evidence for insulin resistance improvement by magnesium supplementation.

**Materials and Methods:**

Rat model of type 2 diabetes was developed using a high-fat diet along with low dose streptozotocin (STZ) treatment. Magnesium supplement was given orally by mixing with the high-fat diet. Serum insulin level, insulin sensitivity, and insulin receptor affinity were assessed using radioimmunoassay (RIA). Insulin receptor, insulin receptor substrate (IRS-2), and β-Arrestin-2 gene and protein expression levels were measured using immunohistochemistry and RT-PCR. Xanthine oxidase assay, thiobarbituric acid reactive substance assay (TCA method), colorimetric assay, and ELISA were used to determine the serum SOD, MDA, T-AOC, and ox-LDL levels, respectively.

**Results:**

Magnesium supplementation enhanced insulin sensitivity and decreased insulin resistance in diabetic rats mainly through increasing insulin receptor expression, affinity, and augmenting insulin receptor signaling. Magnesium supplementation also inhibited lipid peroxidation in diabetic rats and protected against pancreatic cell injury in diabetic rats. In addition, we found that β-arrestin-2 gene expression was suppressed in diabetes, which was possibly attributed to gene methylation modification, as β-arrestin 2 promotor was rich in methylation-regulating sites. Magnesium supplementation could affect β-arrestin-2 gene expression and methylation.

**Conclusion:**

Magnesium supplementation has a positive effect on insulin receptor activity and insulin sensitivity in type 2 diabetes.

## Introduction

Magnesium is an abundant mineral in the human body that functions as a structural component of enzymes and a catalytic co-factor, which plays an important role as a calcium antagonist (1). It is required for the synthesis of DNA, RNA, and the antioxidant glutathione and contributes to the structural development of bones (2). Magnesium is a cofactor in more than 300 enzyme systems that regulate diverse biochemical reactions in the body, including protein synthesis, blood glucose control, blood pressure regulation, and muscle and nerve functions (3). Magnesium is also required for energy production, oxidative phosphorylation, and glycolysis (4). 

Diabetes mellitus (DM) comprises a group of metabolic diseases characterized by hyperglycemia resulting from defects in insulin secretion, insulin action, or both (5). Type 2 diabetes mellitus, previously referred to as noninsulin-dependent diabetes, or adult-onset diabetes, encompasses individuals who have insulin resistance (IR) and usually have relative insulin deficiency (6). IR is a pathological situation characterized by impairment of insulin-mediated glucose transport to peripheral cells, which is due to the lack of physiological response of peripheral tissues to insulin action and leads to the metabolic and hemodynamic disturbances known as metabolic syndrome (7). Increasing attention has been given to the role of certain elements in the pathogenesis of diabetes mellitus, and recent studies have demonstrated the participation of minerals in glucose metabolism disorders in humans (8). Magnesium participates directly in this process by acting as a cofactor for many enzymes involved in energy metabolism and modulating insulin secretion and action in target tissues through interaction with receptors of this hormone (9, 10). Diabetes mellitus has been suggested to be the most common metabolic disorder associated with magnesium deficiency, having 25% to 39% prevalence (5). There are many causes for low magnesium levels in diabetes, which include low magnesium diet, osmotic diuresis, which leads to high renal excretion of magnesium, insensitivity to insulin, which affects intracellular magnesium transport and causes increased loss of extracellular magnesium, usage of loop and thiazide diuretics, which promote magnesium wasting, diabetic autonomic neuropathies, and reduced tubular reabsorption due to IR (11).

Many experimental models for diabetes were developed, which include surgical procedures, chemical induction, and the use of spontaneous or genetically derived animal strains. Chemical methods of inducing damage to the pancreatic β-cells are obtained through the administration of drugs such as streptozotocin (STZ), a nitrosourea derivative isolated from *Streptomyces achromogenes *(12). β-cell toxicity and diabetogenic properties of STZ are mediated through diverse mechanisms including targeted uptake of STZ in β-cells by Glut2 receptors and increased oxidative stress due to nitric oxide release and reactive oxygen species production (13). At the appropriate doses, these drugs act by selectively destroying the pancreatic β-cells (12, 14).

Insulin receptor is a large transmembrane protein, which consists of two alpha chains and two beta chains connected by disulfide bonds (15). During the action state of insulin, it first binds to the insulin receptor causing structural change in the insulin receptor alpha chain to release the inhibition towards the beta chain. Then the beta chain autophosphorylates the tyrosine kinase domains for downstream signaling activation (16). Changes in the intracellular magnesium concentration can regulate the insulin receptor at the gene transcriptional and translational levels by modulating DNA and ribosome structures (17). Alternative splicing occurs during transcription of the insulin receptor gene, which produces two types of mRNAs that translate into high affinity or low-affinity insulin receptors. There are fewer high-affinity insulin receptors but they bind to insulin with high specificity and efficiency. Meanwhile, there are more low-affinity insulin receptors, which have larger capacity to bind to insulin with lower affinity but long-lasting effect. Insulin receptor affinity represents the insulin receptor binding capacity, which is indicated by the binding constant (15, 18). Studies have shown that tissue insulin receptor affinity is significantly down-regulated in insulin-resistant diabetic patients (19).

Currently, it has been shown that elderly type 2 diabetes patients have relatively low serum magnesium levels, which correlates with reduced insulin sensitivity (19). In this study, we used the high-fat diet and STZ-induced rat model of type 2 diabetes to examine the effect of magnesium supplementation on insulin sensitivity. 

## Materials and Methods

***Animals***

Sixty 12 month old, gender and weight-matched Sprague Dawley (SD) rats were used in this study. Animals were housed at a room temperature of 23-25 ^°^C and 12 hr light/12 hr dark cycle and free access to water and rat chow. All procedures of this research were approved by the Ethical Committee of PLA General Hospital.

***Type 2 diabetic rat model***

High-fat diet along with low dose STZ intraperitoneal (IP) injection were used to induce type 2 diabetes in rats as described previously (20). High-fat diet was composed of 10% sugar, 26% lard, 52% L-485, 10% egg yolk powder, 0.3% bile salt, 0.05% dietary fiber, and 1% minerals, with 5.30 kcal/g. Fifty SD rats were fed the above high-fat diet for 60 days followed by IP injection of 25 mg/kg STZ. Ten age and weight-matched control SD rats were fed a normal diet for 60 days followed by IP injection of saline. One week after STZ injection, blood was collected from all animals and the fasting blood glucose level was measured using a Roche blood glucose meter. Radioimmunoassay was used to determine the serum insulin level. Fasting blood glucose level higher than 10 mmol/L was considered to be successful, and the success rate was 80%. Magnesium oxide was purchased from the Kelong chemical reagent factory (1309-48-4, China). Magnesium supplement was applied orally by mixing with the high-fat diet for 4 weeks after successfully modeling diabetic rats. The low-, medium-, and high-magnesium dose groups were given high-fat diets containing 200 mg/kg, 1 000 mg/kg, and 2 000 mg/kg of magnesium, respectively.

***Radioimmunoassay***

A single-cell suspension of erythrocytes or hepatocytes was prepared. Briefly, the rats were starved for 12 hr. Blood was drawn from the abdominal aorta of each rat, and centrifuged at 4 ^°^C, 4000 g, for 20 min. The plasma was separated through the siphon principle. The preservation solution was added to the concentrated red blood cells and thoroughly mixed to form erythrocytes suspension. To prepare hepatocyte suspension, the fresh liver isolated from rats was washed once with 1640 medium, placed in a small dish, gently grounded with a syringe needle, and washed with RPMI-1640 medium while grinding, until the tissue was finished or only a small amount of fiber remained. The liver tissue suspension was collected through a 300 mesh nylon mesh, centrifuged at 1,500 rpm/min for 5 min, and the cells were resuspended in RPMI-1640 medium for use. The cell numbers were determined. 0.0128, 0.0384, 1.152, 3.456, 10.368, 31.104, 93.312 μg of insulin standard was added to non-specific binding tubes, after mixing thoroughly, 100 μl of 125I-insulin and 100 μl of erythrocyte or hepatocyte suspension was added to each tube and kept at 4 ^°^C for 24 hr. Next, 200 μl of separating solution was added and the samples were centrifuged at 3500 rpm/min at 4 ^°^C to remove the supernatant. Radioactivity from the pallets was measured by the automated radioimmunoassay system. The Scatchard software package was used for data analysis and the high-affinity insulin receptor binding constant (K_1_) and capacity (Q_1_), as well as the low-affinity insulin receptor binding constant (K2) and capacity (Q2), were determined. The insulin receptor number (R) was calculated by dividing Q_1_ or Q_2 _values by the erythrocyte or hepatocyte numbers.

***Histology***

Tissue specimens were collected, fixed in 4% paraformaldehyde and embedded in paraffin. For insulin receptor protein staining, skeletal muscle and pancreatic tissue sections (4 µm) were mounted onto glass slides, followed by standard immunohistochemistry staining, which includes deparaffinization, antigen retrieval, blocking, primary and secondary antibody staining for insulin receptor protein (ab40782, Abcam lnc., Cambridge, MA, USA), SABC incubation, and DAB development. Histology photos were analyzed using the Image-Pro Plus software package for optical density quantification of insulin receptor protein level. For measuring pancreatic tissue pathology, hematoxylin and eosin (H&E) staining was performed according to guidelines for clinical samples. 

***RNA isolation and quantitative real-time PCR***

Liver tissues were homogenized in TRIzol reagent. RNA isolation was performed with the RNA Mini-Prep kit according to the manufacturer’s instructions. cDNA was synthesized using Moloney Murine Leukemia Virus Reverse Transcriptase (M-MLV) for reverse transcription. PCR was then performed using TaqMan DNA polymerase with specific primers synthesized by the Shanghai Biotechnology Company (Table 1). PCR setting was first held at 95 ^°^C for 2 min, then 95 ^°^C, 20 sec, 59 ^°^C, 25 sec, and 72 ^°^C, 30 sec for 45 cycles, and 72 ^°^C extension for 5 min. PCR products were analyzed with 2% agarose gel electrophoresis and MUVB.20 system. Relative mRNA levels were calculated based on housekeeping gene GAPDH. 

***Serum SOD, MDA, T-AOC, ox-LDL measurement***

Blood samples were centrifuged at 3500 rpm, 4 ^°^C for 30 min to collect serum. Serum superoxide dismutase (SOD) level was measured using an Amplex™ Red Xanthine/Xanthine Oxidase Assay Kit (A22182, ThermoFisher, USA). The malondialdehyde (MDA) level was measured using the Thiobarbituric Acid Reactive Substances (TCA) method using the OxiSelect™ TBARS Assay Kit (STA-330, Cell Biolabs, USA). Total antioxidant capacity (T-AOC) level was detected using the Colorimetric Assay Kit (E-BC-K219-M, Elabscience). Oxidized low-density lipoproteins LDL (Ox-LDL) was measured using the Ox-LDL ELISA kit (HUIJIA Biotechnology, EHJ-10047, China). All these assays were performed using commercially available assay kits following the manufacturers’ protocols.

***Measurements for serum magnesium, fasting-blood glucose, and serum insulin levels***

The rats were starved for 12 hr, then we collected 3 ml of blood from the abdominal aorta, which was collected in previously labeled polypropylene tubes. The samples were centrifuged at 3500 rpm, 4 ^°^C for 30 min for the collection of serum. Serum magnesium was determined using a Single flame atomic absorption spectrophotometer (AA-3600F, METASH, China). Serum insulin levels were measured using radioimmunoassay. Specifically, radioactive antibody was added to 0.1 ml of serum, incubate at 37 ^°^C then centrifuged, and the pellet radioactivity was measured. Log-logit standard curve was drawn to calculate the insulin levels. Insulin resistant index (ISI)=Fasting serum insulin level x Fasting blood glucose level/22.5, and insulin sensitivity index (ISI)=ln (1/(Fasting serum insulin level×Fasting blood glucose level)). 

***Glucose infusion rate (GIR)***

All hyperinsulinaemic-euglycaemic clamp studies were started after rats recovered from anesthesia (about two hours after surgical procedure). To determine whether IR was present in septic rats, every rat in each group underwent the hyperinsulinaemic-euglycaemic clamp test. Insulin (Novo Nordisk, Tianjin, China) was infused continuously by microinfusion pumps (Terumo, Tokyo, Japan). The insulin dose was started at 10 mU/kg/min. When blood glucose concentration was down to 5.0–6.0 mmol/l, the insulin infusion dose was decreased to 5 mU/kg/min and a glucose infusion was started. Blood glucose concentrations were tested every 10 mins and the glucose infusion rate was modified to maintain basal blood glucose concentration (about 5.0 mmol/l) at the steady-state. IR was expressed by the average glucose infusion rate (GIR; mg/kg/min) for the last 60 mins. GIR was calculated as described in reference 21 using the following formula: GIR=5 mg/kg.min×0.52 kg→2.6 mg/min×60 min/hr÷100 mg/ml→1.56 ml/hr. 

***Statistics***

Data from single comparisons were compared using a two-tailed Student’s t-test, and data for multiple comparisons were compared using a two-way ANOVA as specifically indicated in the figure legends. All statistical analysis was performed using the Prism software package (GraphPad). Data are presented as mean±standard error of the mean (SEM) and values of *P*<0.05 were considered statistically significant.

## Results

***Magnesium supplementation increases insulin receptor affinity in diabetic rats by increasing erythrocyte and hepatocyte insulin receptor binding constant and capacity***

For erythrocytes, high dose magnesium supplement group had K_1 _and Q_1_ values of 2.12±0.33×10^9^ mol/l and 1.27±0.31×10^14^mol/l, respectively, and K_2 _and Q_2_ values of 2.09±0.33×10^7^ mol/l and 3.96±0.41×10^15^mol/l, respectively, which were all significantly higher compared with the diabetic untreated control group (Table 2). To calculate R on the erythrocytes, we divided Q_1_ or Q_2 _values by the erythrocyte number (RBC), which had no significant differences among the groups. R_1_ and R_2_ values for the high dose magnesium supplement group were 82.35±0.77 and 2015.7±62.5, respectively, which were both significantly higher compared with diabetic untreated control group (Table 3). For hepatocytes, high dose magnesium supplement group had K_1, _K_2,_ Q_1, _and Q_2_ values of 4.83±0.09×10^8^ mol/mg (protein), 1.12±0.17×10^6^ mol/mg (protein), 4.46±0.16×10^10^ mol/mg (protein), and 8.32±0.27×10^13^ mol/mg (protein), respectively, which were all significantly higher compared with the diabetic untreated control group (Table 4).

***Magnesium supplementation increases insulin receptor expression in diabetic rats***

**Figure 1:**
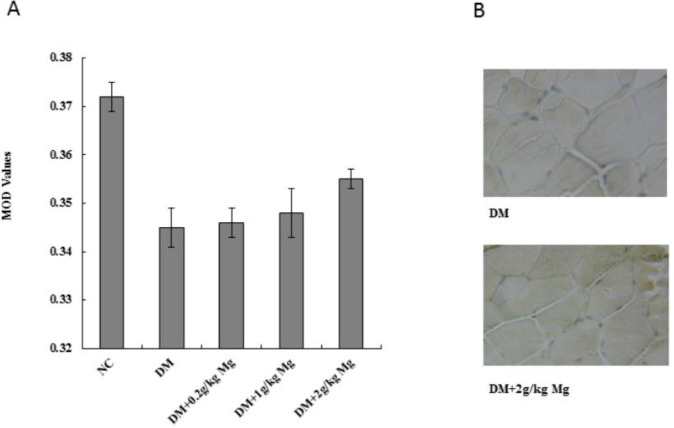
Insulin receptor protein expression levels on skeletal muscle cells after different doses of magnesium supplement treatment. SABC immunohistochemical (IHC) staining was performed on rat quadriceps femoris, and the insulin receptor protein was stained in brown particles. (A) Mean optical density (MOD) values were calculated for each group using Image-Pro Plus software. NC, normal control. DM, diabetic control. Mg, magnesium supplement. (B) Representative IHC pictures from DM group, and DM supplement with 2 g/kg magnesium group. *P<0.05

**Figure 2:**
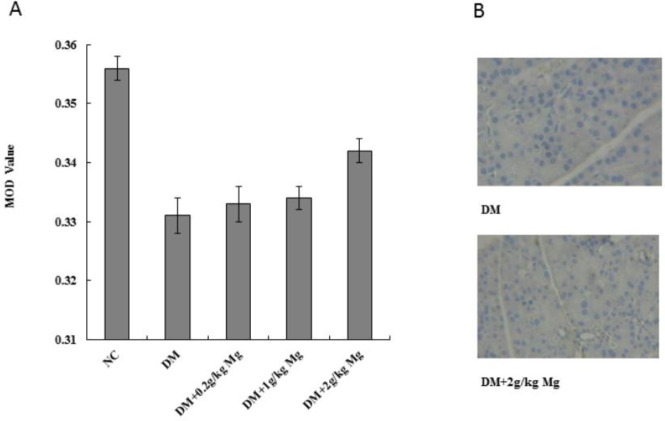
Insulin receptor protein expression levels on pancreatic cells after different doses of magnesium supplement treatment. SABC immunohistochemical (IHC) staining was performed on rat pancreatic cells, and the insulin receptor protein was stained in brown particles. (A) Mean optical density (MOD) values were calculated for each group using Image-Pro Plus software. NC, normal control. DM, diabetic control. Mg, magnesium supplement. (B) Representative IHC pictures from DM group, and DM supplement with 2 g/kg magnesium group. *P<0.05

**Figure 3.**
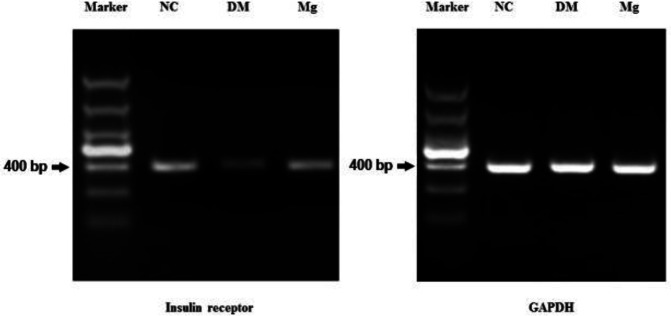
Insulin receptor mRNA levels on hepatocytes after magnesium supplement treatment. RNA was isolated from rat liver tissue and reverse transcribed into DNA. Then PCR was perform using primers specific for insulin receptor gene and house keeping GAPDH gene. Gel electrophoresis was performed to compare the PCR products from each group. NC, normal control. DM, diabetic control. Mg, magnesium supplement. The size of marker from top to bottom are 1500, 1000, 800, 600, 400, 200, and 100 bp

 To determine the insulin receptor expression levels, we used the same diabetic rat model and magnesium supplement treatment, then harvested the pancreas, liver, and skeletal muscle (quadriceps femoris) tissues for analysis. SABC immunohistochemistry staining was performed on pancreas and quadriceps femoris tissues to measure the insulin receptor protein levels, while mRNA was isolated from the liver tissue and the RT-PCR analysis was performed to measure the insulin receptor message levels. The insulin receptors were stained as brown particles by SABC, and we found that the pancreas and the skeletal muscle tissues from the diabetic rats had less staining with lower mean optical density (MOD) values compared with the normal rats. After magnesium supplementation, with high dose, we found a significantly increased level of insulin receptor MOD value (0.355±0.004) compared with the untreated diabetic rat MOD (0.345±0.003) in skeletal muscle tissues (Figure 1). Similarly, high dose magnesium was also able to significantly increase the insulin receptor MOD value in pancreatic tissue (0.342±0.002) compared with untreated diabetic rat MOD (0.331±0.003) (Figure 2). The RT-PCR result from the liver tissue also showed that diabetic rats had lower mRNA levels of insulin receptor compared with the normal rats, and magnesium supplement restored the insulin receptor level in diabetic rat liver (Figure 3).

***Magnesium supplementation promotes insulin receptor substrate (IRS-2) and β-Arrestin-2 gene expression***

**Figure 4:**
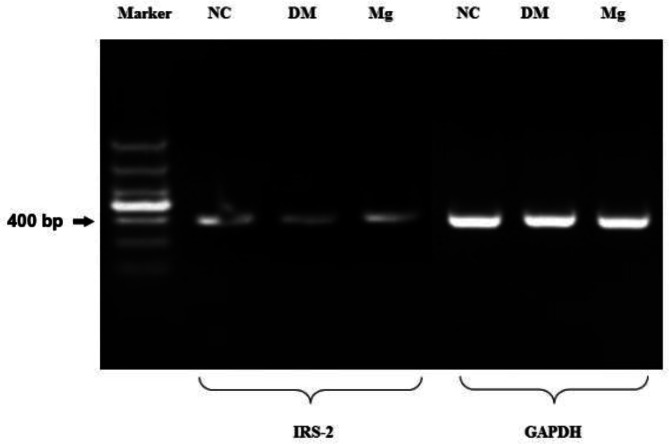
Insulin receptor substrate-2 mRNA levels on hepatocytes after magnesium supplement treatment. RNA was isolated from rat liver tissue and reverse transcribed into DNA. Then PCR was perform using primers specific for substrate-2 (ISR-2) and house keeping GAPDH gene. Gel electrophoresis was performed to compare the PCR products from each group. NC, normal control. DM, diabetic control. Mg, magnesium supplement. The size of marker from top to bottom are 1500, 1000, 800, 600, 400, 200, and 100 bp

**Figure 5:**
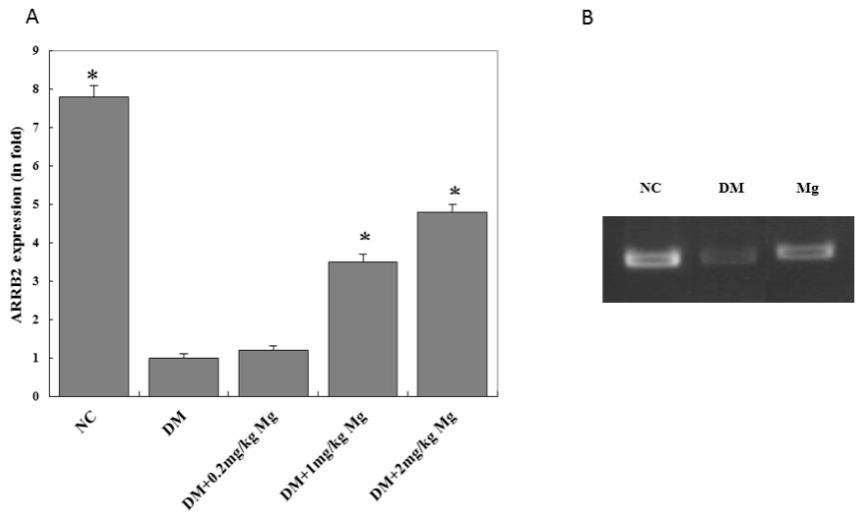
β-Arrestin-2 mRNA levels on hepatocytes after different doses of magnesium supplement treatment. RNA was isolated from rat liver tissue and reverse transcribed into DNA. Then PCR was performed using primers specific for β-Arrestin-2 and house keeping GAPDH gene. Gel electrophoresis was performed to compare the PCR products from each group. (A) Quantitative analysis of β-Arrestin-2 relative fold induction levels. (B) Representative picture of gel electrophoresis result. NC, normal control. DM, diabetic control. Mg, magnesium supplement. *P<0.05

The IRS-2 mRNA level was down-regulated in the diabetic rats compared with the normal rats, and high dose magnesium supplementation increased the IRS-2 mRNA level in the diabetic rats (Figure 4). Similarly, the β-Arrestin-2 mRNA level was also decreased in the diabetic rats, and high dose magnesium supplementation was able to significantly increase the β-Arrestin-2 mRNA level (Figure 5). 

***Magnesium supplementation reduces the oxidative stress in diabetic rats***

**Figure 6.**
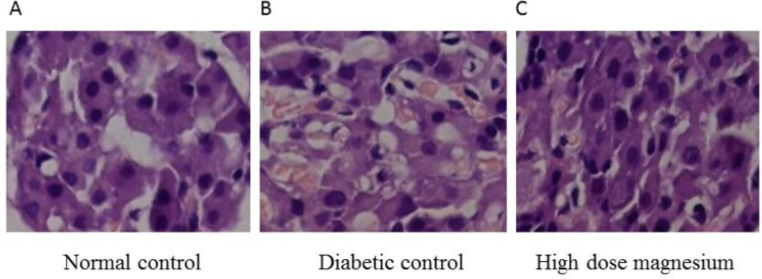
Hematoxylin and eosin stain of pancreatic tissues. (A) Normal control. (B) Diabetic control. (C) High dose (2000 mg/kg) magnesium supplement

Diabetic rats present lower serum levels of SOD and T-AOC compared with the normal rats, and high dose magnesium supplementation significantly increased these levels. Meanwhile, the diabetic rats showed higher serum levels of MDA and ox-LDL compared with normal rats, and high dose magnesium supplementation significantly reduced these levels (Table 5). Using histology, we observed that compared with the normal rats, the pancreatic tissue of the diabetic rats had shrinkage of the islet and reduced islet numbers with irregular shape and border. Also, there were fewer and more scattered islet cells with different shapes, increased cytoplasmic vacuoles, pyknosis, karyolysis, and larger intercellular space. After high dose magnesium supplementation, the pancreatic tissue returned to a more normal state with increased islet size and more islet cells inside that had regular alignment (Figure 6). These results indicate that magnesium supplementation in diabetic rats can reduce the lipid oxidization level and damage to the pancreatic cells. It also enhances the anti-oxidation and proliferative activity of the pancreatic cells, which are crucial for maintaining the pancreatic structure and function, and particularly important for the regulation of insulin levels.

## Discussion

IR as the main feature of type 2 diabetes is a common metabolic abnormality in the elderly population. Animal studies have suggested that a low magnesium diet can result in IR. Magnesium is one of the most abundant ions present in living cells and its plasma concentration is remarkably constant in healthy subjects. Insulin is one of the most important factors that tightly regulate plasma and intracellular magnesium concentrations. Insulin may modulate the shift of magnesium from extracellular to intracellular space, while the intracellular magnesium is also effective in modulating insulin actions, mainly the oxidative glucose metabolism. Studies have shown that poor intracellular magnesium concentration may result in a defective tyrosine-kinase activity at the insulin receptor level and exaggerated intracellular calcium concentration (17). High magnesium intake has been shown to improve glucose metabolism in diabetic patients and stabilize insulin levels (22). However, the cellular and molecular mechanisms of magnesium supplementation in reducing IR and improving glucose metabolism have not been well established.

In this study, we have successfully developed a rat model of type 2 diabetes using a high-fat diet in combination with STZ-treatment. Our results have shown that magnesium supplementation in type 2 diabetic rats increased insulin receptor affinity by increasing erythrocyte and hepatocyte insulin receptor binding constant, binding capacity, and insulin receptor expression levels. Magnesium also promoted insulin receptor substrate (IRS-2) and β-Arrestin-2 gene expressions and reduced the oxidative stress in diabetic rats. Insulin receptor (InsR) and insulin receptor substrate (IRS-2) are key molecules during insulin signaling transduction in the liver. IRS-2 is mainly expressed in hepatocytes and pancreatic β cells, which primarily promotes hepatic glycogen synthesis and inhibits glycogen secretion (23). Recent studies have revealed that β-Arrestin-2 modulates cellular apoptosis by inhibiting Akt activity (24). These results suggest that magnesium supplementation can improve the insulin signaling transduction defect in diabetes.

The high-affinity insulin receptor binding constant (K_1_) and the low-affinity insulin receptor binding constant (K_2_) are the main parameters representing insulin receptor affinity. Reports showed that type 2 diabetic patients have lower K_1_ and K_2_ values, which can be restored by glutamine supplementation in type 2 diabetic rats (25, 26). In our study, we have shown that magnesium supplementation can also increase K_1_ and K_2_ values compared with the control group, which indicates improved insulin receptor affinity. This is consistent with the reports showing magnesium supplementation in type 2 diabetic patients decreases blood glucose level and improves IR. 

Q_1_ and Q_2_ values represent high and low-affinity insulin receptor capacity while R_1_ and R_2_ values represent high and low insulin receptor numbers. When the insulin receptor capacity decreases in the target cells, this suggests that the cell surface insulin binding number is lower in this target tissue when there is the same level of insulin in the serum, which means the effectiveness of insulin is reduced. Lower R_1_ and R_2_ values suggests there are low insulin receptor numbers, which means that even when there’s plenty of insulin in the system, it’s biological functions are diminished. Studies have shown that insulin receptor capacity decreases during IR (27). In our study, the diabetic control group had significantly lower R_1_ and R_2_ values compared with the normal control group. After high dose magnesium supplementation, the diabetic group showed significantly increased R values, which indicates that magnesium can promote the recovery of insulin receptor capacity in type 2 diabetic rats. 

Insulin receptor affinity is the comprehensive representative for the binding capacity of insulin with the insulin receptor. The insulin receptor binding constant (K) is the basic standard for insulin receptor affinity which corresponds to the biological activity of the insulin receptors. High-affinity binding constant (K_1_) indicates the glucose metabolism capacity during stress state or high-intensity physical exercise, meanwhile, the low-affinity binding constant (K_2_) indicates insulin receptor activity and storage capacity during the steady state. The insulin receptor binding capacity (Q) represents the total count of insulin receptor binding sites, which directly affects the insulin binding numbers with the receptor. The high and low-affinity insulin binding capacity (Q_1_ and Q_2_) indicate the total number of insulin receptor binding sites during active and steady-state, respectively (28). Our results show that diabetic rats have lower values of K_1_, K_2_, Q_1_, Q_2_, R_1_, and R_2_ compared with the normal rats, and high dose magnesium supplementation can significantly increase these values, which suggests that magnesium supplementation can enhance insulin affinity in diabetic rat erythrocytes and hepatocytes. In our previous clinical study with elderly diabetic patients, we also found that magnesium supplementation increased erythrocyte K1, K2, Q1, Q2, R1, and R2 values (data not published). Therefore, both animal and human studies indicate the effect of magnesium supplementation in enhancing insulin receptor activity.

The insulin regulation of glucose metabolism is through insulin receptor expression on the cellular membrane. The liver is one of the major organs for glucose metabolism, while skeletal muscles are the major tissues for energy generation by glucose metabolism (29). Therefore, the insulin receptor expression levels on the liver and skeletal muscle cells can affect the active insulin numbers and the glucose metabolism level. Moreover, the insulin receptor expression level on pancreatic cells serves as a positive feedback mechanism for insulin secretion (30). Our results suggest that insulin receptor expression levels were decreased in the liver, skeletal muscles, and pancreatic cells in diabetic rats compared with the normal rats, and high dose magnesium supplementation can restore the insulin receptor expression levels in diabetic rats, which plays an important role in increasing insulin sensitivity and insulin receptor affinity.

MDA is the end product of lipid peroxidation, which can cause various damages to the cell membrane, phospholipids, proteins, nucleic acid, and enzymes (31). The diabetic patients are under high oxidative stress, due to the increase of oxidation products and the decreased clearance ability for free radicals, which lead to a series of lipid peroxidations and the accumulation of the end products (32). In this study, we found that there was a higher MDA level in the diabetic control group compared with the normal group, while high dose magnesium supplement group had reduced levels of MDA compared with the diabetic control group, which suggests that magnesium supplementation has an inhibitory effect on lipid peroxidation in diabetic rats. The reduction in lipid peroxidation product can help protect the body from the damage by free radicals, keep the integrity of the cell membrane, and maintain the normal function of the cells. Since the insulin receptor is a type of cell membrane receptor, the integrity of the cell membrane structure is crucial for the effective biological functions of the insulin receptor.

Interestingly, we have also discovered that diabetic rats had reduced RNA levels of downstream signaling molecule β-Arrestin-2 compared with the normal rats, which was potentially associated with gene methylation. Magnesium supplementation could restore the β-Arrestin-2 level in diabetic rats. Further gene sequence analysis (33) uncovered that the promoter region of β-Arrestin-2 consisted of highly concentrated methylation sites (supplementary figure), which supported our hypothesis. Currently, there is no publication describing β-Arrestin-2 gene methylation in association with diabetes. Under normal conditions, β-Arrestin-2 protein forms a signaling complex with the insulin receptor, with the β-Arrestin-2 being the core component of this complex, which couples the upstream insulin receptor with the downstream signaling kinases. A decrease in β-Arrestin-2 protein level or functional deficiency can lead to the inhibition of the signaling transcription which results in IR in diabetes (34, 35). Thus, understanding the methylation modification in regulating the β-Arrestin-2 gene expression level is highly important for providing new information for the investigation of diabetes etiology, and has great significance in molecular genetic study, as well as early diagnosis and treatment for type 2 diabetes.

**Table 1:** Primers used in quantitative real-time PCR for liver tissue mRNA quantification

Gene	Forward primer	Reverse primer
Insulin receptor	CCGAGAGCTGGGGCAGGGAT	GTAGGGGGAGGACGGCCTGG
ISR-2	GCCTTCGTGCCCACCCACTC	CCACAGCCTTGGCAGCACCA
β-Arrestin-2	GGAGATTTTTGTAGGTTTTGGA	ACCAAAATCCCTACTCCTCC
GAPDH	TCTCCGCCCCTTCCGCTGAT	CCACAGCCTTGGCAGCACCA

**Table 2:** Erythrocyte insulin receptor binding constant and capacity

Groups	K_1_ (×10^9^ mol/L)	K_2_ (×10^7^ mol/L)	Q_1_ （×10^14^ mol/L）	Q_2_ (×10^15^ mol/L)
Normal control Diabetic control Low dose Medium dose High dose	2.59±0.33* 1.17±0.45 1.25±0.46 1.69±0.39* 2.12±0.33 *	2.67±0.41* 1.31±0.49 1.18±0.52 1.62±0.37* 2.09±0.33*	1.69±0.28* 0.91±0.25 1.16±0.33 1.25±0.26* 1.27±0.31*	4.75±0.52 3.04±0.39 3.01±0.43 3.47±0.38 3.96±0.41

Data are means±SD unless otherwise indicated, (n=10).

*Compared with diabetic control group, *P<*0.05

**Table 3:** Erythrocyte insulin receptor numbers

Groups	R_1_	R_2_	RBC （×10^12^/L）
Normal control Diabetic control Low dose Medium dose High dose	87.03 ±0.92* 63.71 ±0.84 64.82 ±0.89 66.51 ±0.95* 82.35±0.77*	2538.7±59.2* 1836.3±58.9 1906.1±75.9 1864.2±66.5* 2015.7±62.5*	1.64±0.32 1.59±0.38 1.61±0.39 1.67±0.42 1.62±0.45

Data are means±SD unless otherwise indicated, (n=10).

*Compared with diabetic control group, *P<*0.05; RBC: Red Blood Cell

**Table 4:** Hepatocyte insulin receptor binding constant and capacity (mol /mg protein)

Groups	K_1_ (×10^8^)	K_2_ (×10^6^)	Q_1_ （×10^10^）	Q_2_ (×10^13^)
Normal control Diabetic control Low dose Medium dose High dose	5.40±0.11* 3.60±0.10 3.72±0.12 4.19±0.39* 4.62±0.33 *	1.44±0.09* 0.76±0.09 0.81±0.11 0.95±0.07* 1.14±0.10*	6.83±0.28* 3.91±0.26 3.96±0.31 4.79±0.28* 5.11±0.30*	10.52±0.42* 6.04±0.32 6.01±0.43 7.25±0.42* 8.06±0.39*

Data are means±SD unless otherwise indicated, (n=10).

*Compared with diabetic control group, *P<*0.05

**Table 5:** SOD, MDA, T-AOC, and ox-LDL levels

Groups	SOD (U/ml)	MDA (nmol/ml)	T-AOC （U/mL）	Ox-LDL (ng/mL)
Normal control Diabetic control Low dose Medium dose High dose	336.58±23.50* 245.70±20.57 249.62±22.25 265.56±28.62 297.76±31.30 *	8.96±0.53* 12.35±0.62 11.86±0.70 11.13±0.67 9.86±0.75*	11.99±1.10* 7.53±1.07 7.46±1.03 9.46±1.51* 9.82±1.17*	14.85±1.43* 24.05±1.27 22.96±1.06 16.47±1.12* 15.04±1.17*

Data are means±SD unless otherwise indicated, (n=10)

*Compared with diabetic control group, *P<*0.05

SOD: Superoxide dismutase, MDA: Malondialdehyde, T-AOC: Total anti-oxidant capacity, ox-LDL: Oxidaized low-density lipoproteins

## Conclusion

Overall, our study suggests that magnesium supplementation has a positive effect on insulin receptor activity and insulin sensitivity in type 2 diabetes by inhibiting lipid peroxidation and up-regulating β-Arrestin-2 gene expression. Therefore, this study provides new insight into the important role of magnesium in regulating metabolism. This could be greatly important for developing a future treatment of type 2 diabetes and related metabolic diseases.
